# InSAR surface deformation and numeric modeling unravel an active salt diapir in southern Romania

**DOI:** 10.1038/s41598-021-91517-4

**Published:** 2021-06-08

**Authors:** Vlad Constantin Manea, Iuliana Armaş, Marina Manea, Mihaela Gheorghe

**Affiliations:** 1 Computational Geodynamics Laboratory, Centro de Geociencias, Universidad Nacional Autónoma de México, Campus Juriquilla, 76230 Querétaro, Mexico; 2 Astronomical Institute of the Romanian Academy, 040557 Bucharest, Romania; 3 Research Institute of the University of Bucharest–iCUB, University of Bucharest, 050095 Bucharest, Romania; 4 Department of Geomorphology-Pedology-Geomatics, Faculty of Geography, University of Bucharest, Nicolae Balcescu 1, Sector 1, 010041 Bucharest, Romania; 5 GMV Innovating Solutions SRL, Calea Floreasca 246C, 077190 Bucharest, Romania

**Keywords:** Geodynamics, Geomorphology

## Abstract

Salt diapirism is often associated with potential hydrocarbon energy resources, and detecting active diapirs can strongly affect the prospect to discover new gas and oilfields. Here we use InSAR techniques as a proxy to evaluate surface deformation in the Diapiric Fold Zone located in the East Carpathians Bend. Significant surface uplift (~ 5 mm/year) is identified in a relatively small region not previously known for the presence of an actively rising salt diapir. Using high-resolution two-dimensional thermomechanical numerical simulations of salt diapirs intrusions, we show that that the observed surface deformation can be induced by a relatively small salt diapir (1–2 km in diameter) rising from an initial salt layer located at < 7 km depth. We constrain the salt diapir viscosity by comparing the InSAR surface deformation pattern with results from numerical simulations and our best fitting model is obtained for a salt viscosity of 1 × 10^17^ Pa s. The best fitting model reveals the presence of a relatively small salt diapir that has not pierced yet the entire sedimentary layer and is located just 1–2 km below the surface.

## Introduction

For more than a century and a half, salt tectonics represented a key research topic closely related with the formation and evolution of major hydrocarbon provinces. One distinct characteristic making salt different from most of sedimentary rocks is the formation of diapirs, which represent a gravitational instability that uplifts as anticlinal or domal structures and discordantly pierces or intrudes the overlying rock layers^1^. Compared with other rocks, salt is also mechanically weak^2^ and less dense than most carbonates and all compacted siliciclastic rocks. Additionally, salt has the property to easily deform and undergo diffusion creep under low strain rates exhibiting linear viscous behavior. Due to this unique combination of properties, salt can easily rise in form of diapirs under specific tectonic stress gradients^3^. Salt tectonics started to become gradually important in oil and gas exploration in the past few decades, where salt domes or diapirs, can successfully long-term trap hydrocarbons due to its extremely low permeability. Its low viscosity (and high ductility^4^) allows the formation of domal shapes which create favorable conditions for trapping hydrocarbons. From a global economic perspective, salt related structures create perfect seals, and a large portion of the Earth’s hydrocarbon reserves are stored in salt traps (e.g. 60% of the Persian Gulf Basin oil fields are related with salt diapirs^5^). Therefore, salt tectonics becomes of high importance in the oil industry, and also for other scientific disciplines^6^. For example, diapir formation can be related to slope instabilities and removal of the overburden in landslide prone-areas, where monitoring active salt domes is important for vulnerability assessments. However, salt diapirs that have not pierced all the way through the surface are often difficult to unravel by cost-effective standard survey techniques.

In the last decades, the global availability of both commercial and free satellite imagery has greatly reduced the costs for high quality monitoring of ground surface deformation. Since 2001, when ref.^7^ proposed the first multi-temporal interferometric technique for calculating displacement time series with millimeters accuracy, Synthetic Aperture Radar Interferometry (InSAR) methods have become a valuable tool in geological studies related with small earth surface deformation over wide areas. InSAR is a modern satellite technique that offers the possibility to detect sub-millimeter surface displacement over very large areas with high temporal and spatial resolution. The main condition for successfully applying the InSAR technique over an area is the presence of radar coherent targets on the observed surface. These targets are usually represented by man-made features, such as buildings and infrastructure, dams and non-vegetated rocks in natural areas. Based on the large variability of tectonic and geological processes that cause surface deformation, the original classic InSAR algorithm suffered different adaptations. Today specifically designed InSAR algorithms are used to detect subsidence in urban and peri-urban areas^8–10^, crustal displacement caused by seismic events^11,12^, volcanic activities^13,14^, slow developing landslides^15–17^, as well as for sinkholes monitoring^18,19^. InSAR was used also to study salt related uplift patterns, associated with zones of transpression and salt diapirism^5,20,21^.

Scaled analogue models are useful in understanding some of the critical key factors governing the tectonic evolution of salt diapirs. For our particular study area only analogue modeling of salt tectonics was recently performed^22^. However, the analogue models have some intrinsic but somehow strict limitations, mostly in terms of realistic rheology^22^. These limitations are circumvented by using advanced numerical modeling techniques. Efforts to numerically investigate two fluid flow regimes date back to the 1967 when marker and cell technique were used to solve the Navier–Stokes equation for a two fluids system^23^. Subsequent numerical studies revealed that when a bottom layer fluid is less viscous than the overburden fluid, bubble or plume shaped structures are formed^24,25^. Other studies used numerical modeling techniques specifically applied for investigating salt diapirism, as for example employing simplified Newtonian viscous fluids^4^. With time, numerical investigations started to employ innovative schemes based on viscoplastic rheology which allows to study large nonlinear deformations, which however are not specific for salt diapirism^26–30^. However, despite their limitations, numerical and analogue modeling proved to be a powerful tool in understanding salt diapir formation and evolution in the context of sedimentary basin tectonics^4,27,30,31^.

**Figure 1:**
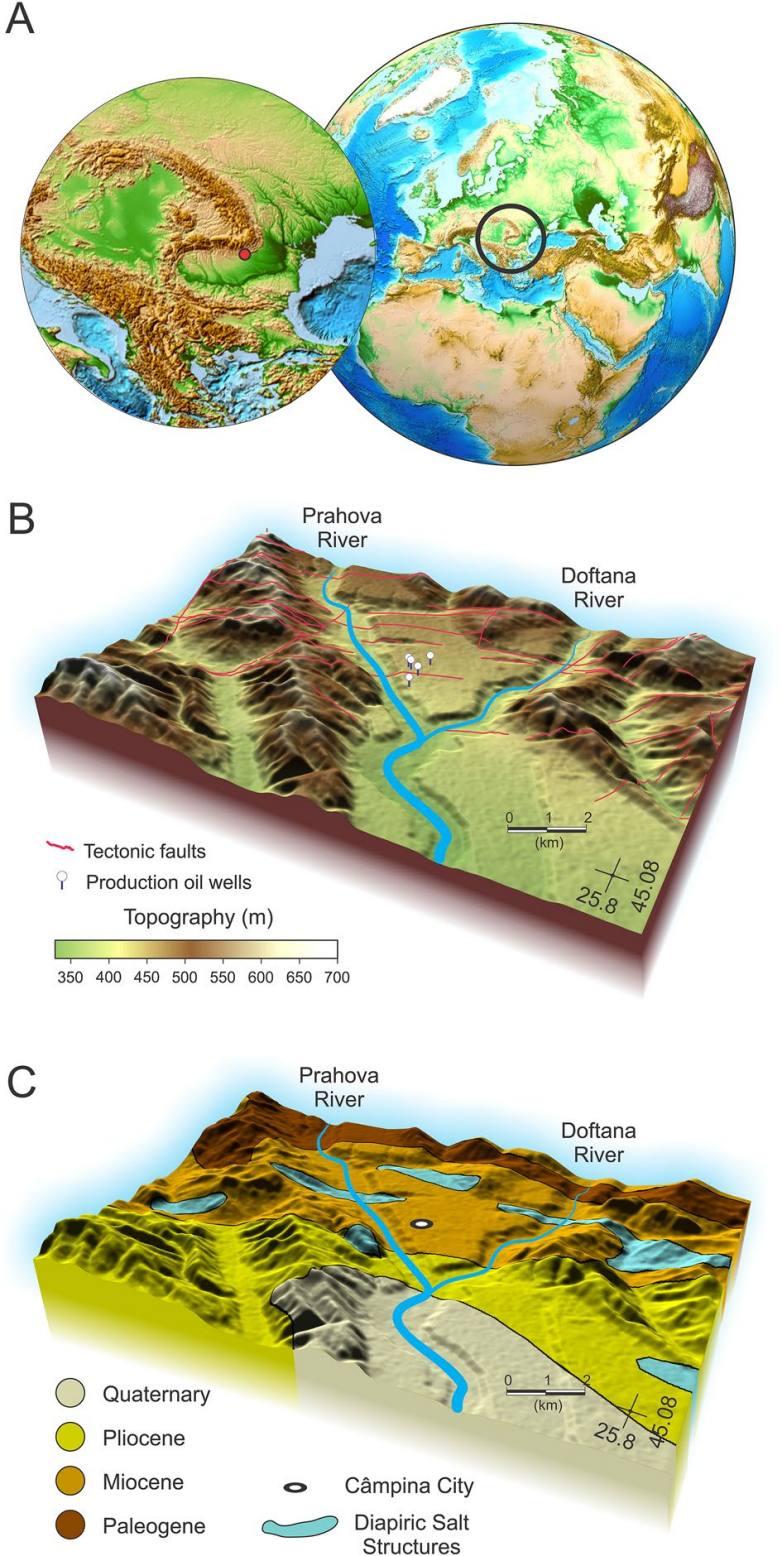
(**A**) Global topography/bathymetry maps showing the location of the regional study area (black circle). Red dot in the left-hand inset marks the position of the study area in the East Carpathians Bend zone. Maps were created based on ETOPO1 Global Relief Model dataset from ref.^32^ and generated with the open-source software ParaView (http://www.paraview.org) version 5.0.1, licensed under the CC BY 4.0 license (https://creativecommons.org/licenses/by/4.0/). (**B**) Color-shaded 3-D view of the topography study area. Red curves draped over the shaded relief represent tectonic faults^33^. White round markers show the location of several production oil wells located in the vicinity of the Prahova and Doftana rives confluence. (**C**) Color-shaded 3-D view of the simplified geological map of the Diapir Fold Zone draped over the topography^22,34^.

In this study, we employ the Small BAseline Subset algorithm (see “Methods” section) for assessing the C-band SAR imagery acquired by the Sentinel-1A and B satellites (Supplementary Table S1) for the Diapiric Fold Zone (DFZ) located in the East Carpathians Bend (Fig. 1), and show for the first time the surface deformation associated with a previously unknown rising salt diapir. Additionally, we employ a high-resolution time-dependent visco-elasto-plastic thermomechanical model (see “Methods” section). Since the salt in the East Carpathians Bend DFZ is Early Miocene, we integrate our numerical simulations since Late Miocene (~ 10 Ma), and taking into account the extensional and compressional regimes during this period. We use the InSAR surface deformation observations associated with the newly discovered active salt diapir to constrain numerically salt viscosity, a rock property that is still not well known^26^. Additionally, this study shows that the combination between InSAR and numerical modeling technique represents a powerful and cost-effective tool for preliminary identification and space–time tectonic evolution investigation of unknown active salt diapirs.

## Study area

The DFZ study area focuses on the orogen-foreland contact, between the localities Telega and Câmpina, in the vicinity of Prahova and Doftana rivers confluence (Fig. 1, Supplemental Fig. S1). From an economic history perspective salt extraction started in Telega around mid-eighteenth century. By the end of the eighteenth century, industrial-scale oil extraction also started in the Câmpina region, actually Romania was the first country in the world to extract oil on an industrial scale, with 275 tons of oil extracted in the 1857-year alone^35^. Actually, oil extraction in this area dates back to Roman Empire times, and in the year 1646 oil was already extract from shallow wells^36^. In 1856 the first oil refinery in the world is built in the city of Ploiesti, located some 30 km south of Campina city (Fig. 1), followed in 1897 by the biggest and most modern oil refinery in Europe built at Câmpina^35^. This might not be surprising, since Romania probable holds the largest salt resources in Europe^37^.

Although debates regarding salt tectonics in Romania started based on previous field observations^38^, the geological term salt diapirism was introduced first by ref.^39^, and provided a fundamental framework for salt related tectonics interpretations worldwide. Some early studies revealed that salt domes located in our study region are confined to the axial portions of sharp anticlinal folds and fracture zones, and the salt started to intrude the overlying sedimentary in late Pliocene (~ 0–2.6 Ma) or even early Pleistocene (~ 1.8 Ma)^40^. In the East Carpathians Bend DFZ, salt is Burdigalian in age (early Miocene)^41^ and the initial salt basin was deformed from its tabular undisplaced position by thrust over the foreland through a succession of five regional deformation stages since early Miocene (~ 20 Ma)^42^. The so-called Lower Miocene (Burdigalian) Lower Salt Formation is composed of more or less massive salt bodies associated with sedimentary breccias, which occasionally replace the salt completely. Seismic-reflection studies^42^ revealed that the salt layer is overlain by a thick (3–4 km) molasse sequence of Early to Mid-Miocene age that consists of sandstones, marls and silts layers alternating with medium- to thin-bedded gypsum and tuffs layers. The post-tectonic relatively thin (< 2 km) Upper Miocene-Pliocene cover units’ formations also include mainly marls, sandstones as well as calcareous sandstones, sand and small coal beds and pebbles. The layer of sediments above the salt layer is confined into a complex system of faults which is mainly oriented subparallel with the South Carpathians fold and thrust belt (Fig. 1)^43^.

The associated folding during the Walachian compressional stage of Pleistocene age^44^ is thought to play an important role in salt displacement upward by lateral shortening and rejuvenated pre-existing diapirs located on the eastern Moesian Plate^42^. The Post-Nappe Emplacement extensional stage (13–5.3 Ma) and the Walachian folding stage (5.3 Ma–present day) exhibit a N-S or NNW-SSE directed compressional stress field documented by detailed microstructural studies^42,45,46^. The total shortening is estimated by ref.^47^ at 130 km, divided in ~ 85% during the mid-Miocene and the rest for the remaining period. Previous studies of salt diapir formation and propagation indicate the formation of relatively thin mushroom type heads that are subsequently squeezed up to their present positions^42^. Interestingly for this study, ref.^48^ considered the diapirs in the DFZ detached from their source layer due to lateral compression. Also, ref.^48^ observed that the sediment layers are oriented subvertical adjacent to the diapir and thinned towards to the diapir top. Since salt diapir upward migration and fault system formation during the tectonic extensional and compressional stages might be coeval processes, the role of the main Campina fault system (Fig. 1) in the formation and subsequent development of salt diapirs remains not well understood.

## Results

### Surface displacements and rising of a new salt diapir

Salt diapirs are specific for the DFZ (Fig. 1C), and several salt diapirs have already burst out from their overburden (Fig. 1C) and formed typical topographic structures (i.e. lakes). In this study we employ InSAR for a spatially limited region centered on the Campina City where several production wells are still extracting crude oil after more than a half a century (Fig. 1). Using the Small-BAseline Subset (SBAS) technique (Supplementary Table S2), we processed two stacks of 123 Sentinel-1 A and B satellite images acquired from both descending and ascending orbit over a 4-year period, between 2014 and 2018, covering the study area (Fig. S2). Optimal temporal and spatial baselines were set for each of the datasets in order to increase coherence of the interferograms (see “Methods” section and Supplementary Information for more details).

After processing approximately 30,000 points were obtained from each image stack with a density of approximately 1200 points/km^2^. For each point, the surface displacement and displacement rates between each image acquisition were derived in the Line of Sight of the satellite. The use of both ascending and descending geometries offers a two-dimensional view of the movement of the earth surface (Supplementary Fig. S1). During ascending pass, the LOS is directed from West to East while during descending pass a target is observed from East to West. Both geometries were combined to derive displacement on two directions: vertical and horizontal East–West displacement.

**Figure 2:**
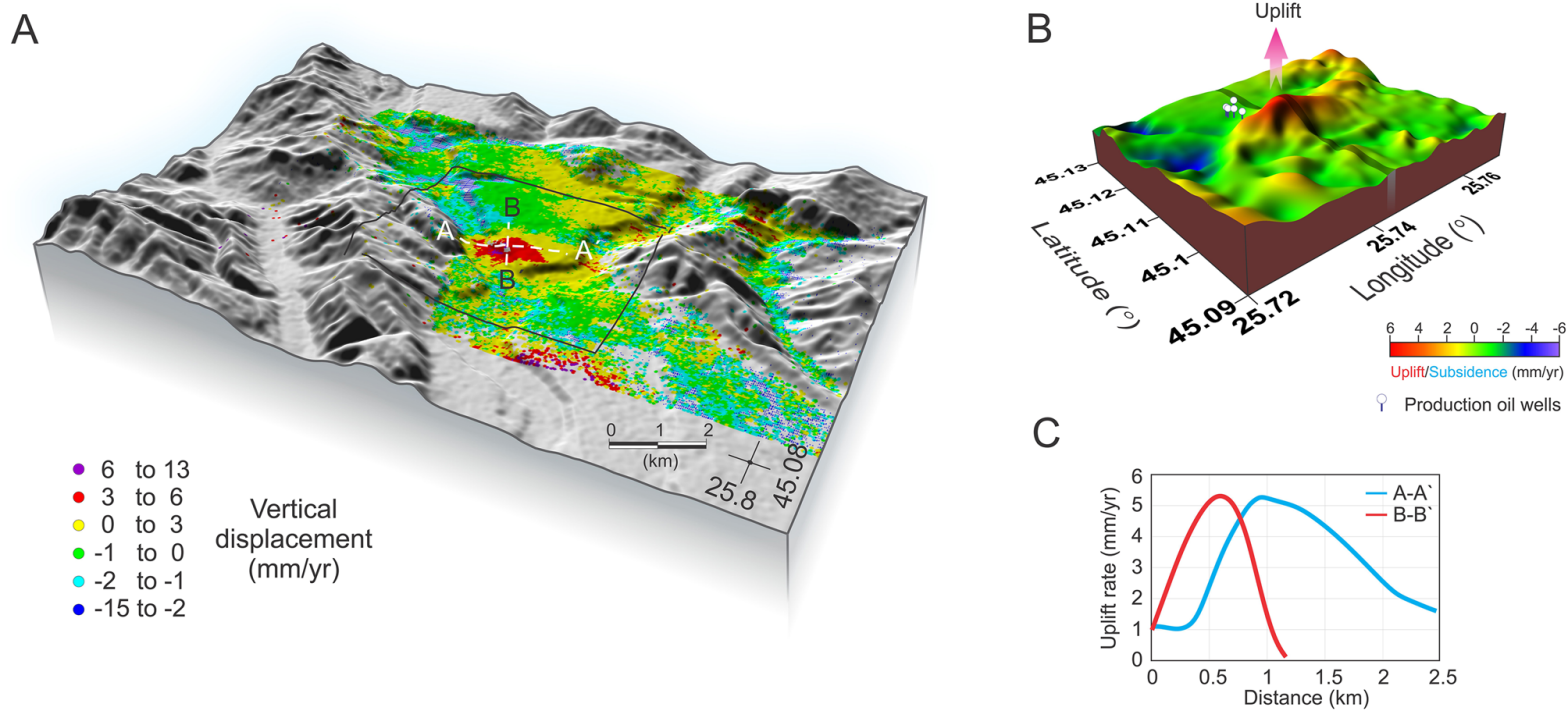
(**A**) InSAR uplift rates draped as data points over the gray-shaded 3-D view of the topography in the study area. The black square shows the location of the 3D view region shown in (**B**). A-A′ and B-B′ represent the two perpendicular cross sections through the maximum uplift region shown in (**C**). (**B**) Color-shaded 3-D view of the InSAR uplift rates. White round markers show the location of several production oilrigs. Red vertical arrow depicts the maximum uplift. The middle N–S gray band shows the location of model cut shown in Fig. 4. 3D maps in (**A,B**) are generated with the open-source software ParaView (http://www.paraview.org) version 5.0.1, licensed under the CC BY 4.0 license (https://creativecommons.org/licenses/by/4.0/). (**C**) Vertical cross-sections A-A′ and B-B′.

Resulted displacement time series over a time span of four years reveal a clear deformation pattern located in the southern part of the Campina city (Fig. 2, Supplemental Fig. S3). Stable areas display velocity values between − 0.5 and + 0.5 mm/year. The velocity values in the study area range from − 20 up to + 6 mm/year. Some points found mainly along the Prahova riverbed are characterized by translational trends that indicate landslides. Whereas other regions exhibit only limited finite surface movement (uplift or subsidence), our study reveals an elliptical shape uplift region with a maximum displacement rate of up to 6 mm/year for the apex area (Fig. 2B,C). In general, salt diapirs that pierced the surface are characterized by both horizontal and vertical movements^5^. The deformation pattern observed in this study is characterized mainly by strong uplift velocity, with relatively low horizontal movement (up to 2 mm/year).

The elliptical uplifted area is not quite centered above the topography summit between the Prahova and Doftana rivers, suggesting probably the existence of a buried salt diapir that is asymmetrically fed. Actually, just some 15 km SW from our study area the seismic-reflection studies revealed the presence of a bottom continuous (TWT ~ 0.5 s) salt layer that feeds several diapirs (Fig. S4). Another important observation is the location of the oil wells on the northern side of the uplifted region, but right behind the active area (Fig. 2B). This might indicate that a potential rising salt diapir creates structural traps able to capture hydrocarbons for long periods of time.

### Numerical modeling of a salt diapir

**Figure 3:**
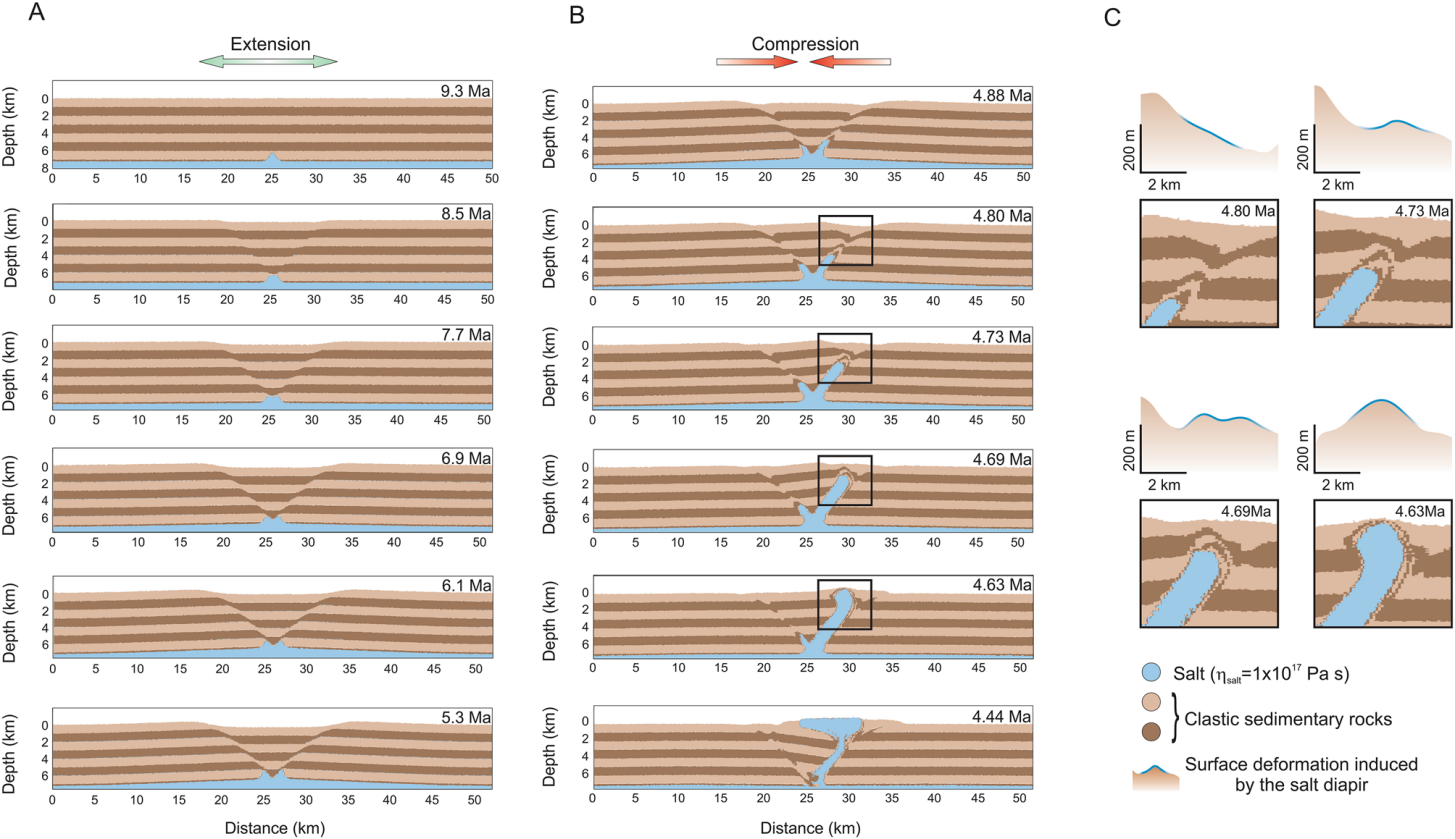
Modeling results of a salt diapir (blue color) piercing through an initial 7.2 km thick sedimentary layer (represented in two brown color tones for better visualization of deformation). In this numerical experiment the initial salt layer thickness is 800 m and salt diapir viscosity of 1 × 10^17^ Pa s. In all numerical experiments we applied horizontal velocities at the lateral boundaries of the model that are equal in size and opposite in direction. Their magnitude is set to 0.5 mm/year (0.25 mm/year applied for each side in opposite divergent direction (extension)) for the first 4 Myr (**A**), followed by 1.0 mm/year (0.50 mm/year applied for each side in opposite convergent direction (shortening)) (**B**). For a domain width of 50 km this results in a background strain rate of + 3.17 × 10^–16^ 1/s and − 6.34 × 10^–16^ 1/s respectively. (**C**) Surface deformation evolution associated with the incoming salt diapir.

To better understand the formation and evolution of a salt diapir in the study region, we developed high-resolution two-dimensional thermo-mechanical simulations to predict temperature, viscosity, density, stresses, surface deformation and rocks spatial distribution and evolution along a 2D profile. The initial geometry of the numerical models (Fig. S5) together with specific model parameters (Supplementary Table S3) are presented in Supplementary Material. Our approach towards evaluating the possibility of an initial buoyant salt layer located at the bottom of our model domain involves a set of two-dimensional numerical models that have lateral boundary conditions in agreement with the compressional Walachian and extensional Post-Nappe Emplacement deformation stages, and are integrated in time since Late Miocene (9.3 Ma). Since most of the post-Oligocene shortening (> 83%) in the Eastern Carpathians is concentrated during the mid-Miocene and the remaining < 17% afterwards^47^, we introduced a shortening background strain rate of 4.76 × 10^–16^–7.93 × 10^–16^ s^−1^ (0.75–1.25 mm/year) which is applied as half shortening rate in opposite direction at each lateral boundary of our 50 km wide model domain for the Walachian stage (Fig. S5). This corresponds to a total shortening consistent with the < 17% reported by ref.^47^. However, in order to qualify the effect of both tectonic shortening and extension on salt diapir formation we also include in our models an extensional background strain rate of 2.85 × 10^–16^–3.49 × 10^–16^ s^−1^ (0.45–0.55 mm/year) corresponding to the extensional Post-Nappe Emplacement stage^42^. The original salt layer thickness before rising in diapir is not well constrained, and ref.^42^ suggest a thickness of several hundreds of meters. Geological cross-sections (Fig. S4) through the DFZ show the existence of a relatively continuous, although deformed, less than 1 km thick salt layer located at depths > 7 km (TWT ~ 5 s)^22,49^. Therefore, in our numerical model we introduced at the bottom of the modeling domain a uniformly 800 m thick salt layer with a small Gaussian shape anomaly in the center-bottom of the modeling domain (Fig. S5). Modeling results are presented in Fig. 3, and additional modeling results and benchmarks can be found in Supplementary Information material (Figs. S6–S9).

## Discussion

**Figure 4:**
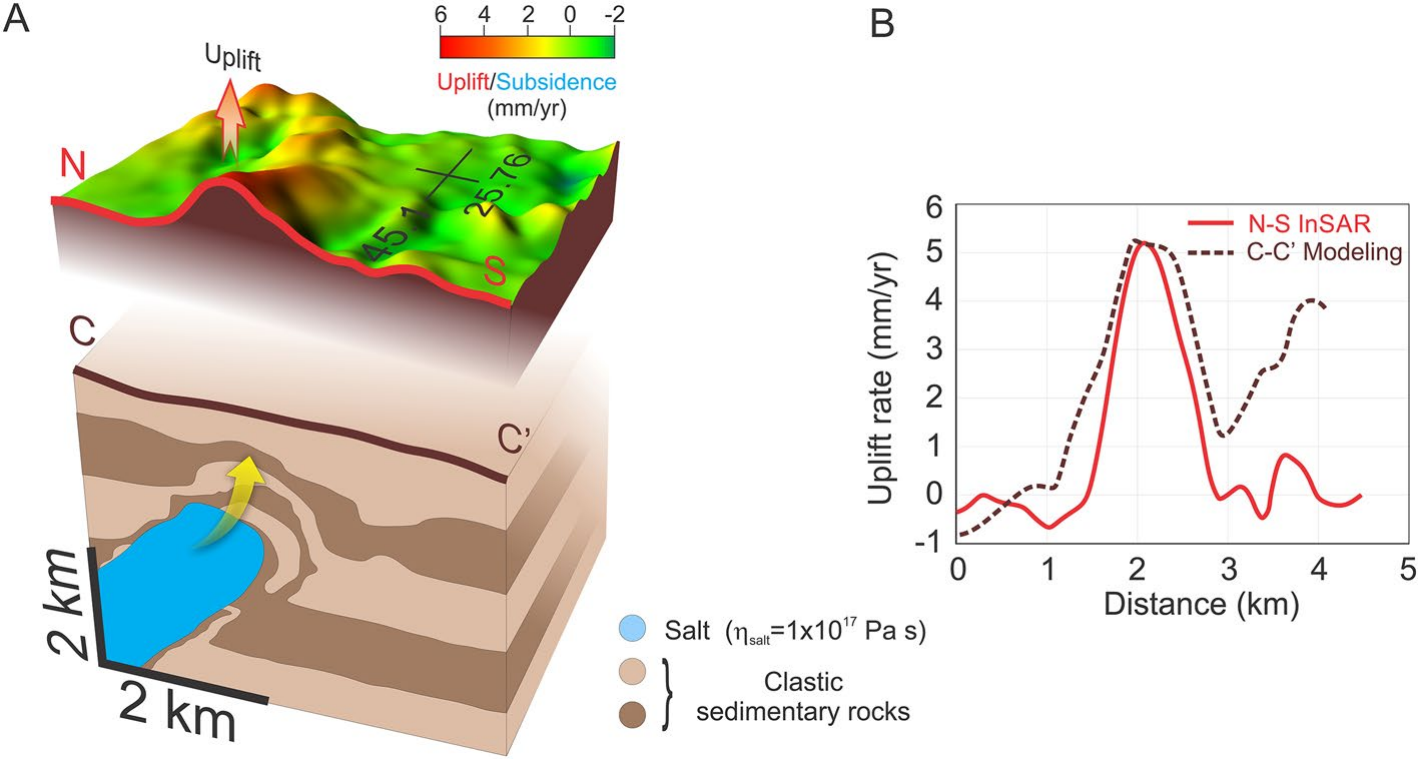
(**A**) Perspective image illustrating the InSAR uplift pattern (top) and the result from numerical simulation (shown in Fig. 3) where salt diapir (blue color) intrudes (yellow arrow) the overlying sedimentary rocks. 3D top map is generated with the open-source software ParaView (http://www.paraview.org) version 5.0.1, licensed under the CC BY 4.0 license (https://creativecommons.org/licenses/by/4.0/). (**B**) N-S cross-section of InSAR uplift rate vs. maximum surface uplift inferred from the numerical simulation where salt diapir viscosity is considered 1 × 10^17^ Pa s.

It is known that salt diapirs can strongly affect the surface topography once they approach the surface. Uncovering regions that experience concentrated uplift can likely indicate the presence of rising salt diapir. The DFZ in Romania is a good place to track and explore such effects due to the presence of a large number of salt diapirs that have already pierced the overburden sedimentary layer to the surface (Fig. 1C). Although the present study region is limited to a small part within the DFZ where we evaluate spatial–temporal variations in deformation using InSAR, extending this research to other areas would be an interesting research topic to pursue in the future. Using InSAR techniques, we can estimate the magnitude of surface uplift without a detailed knowledge of actual subsurface geology and tectonic processes both of which vary from region to region. In this study we reveal significant and concentrated uplift rate (~ 5 mm/year) in a DFZ region previously unknown for the presence of a salt diapir (Fig. 2). This is best explained in terms of a currently rising salt diapir that has not pierced the overlying sedimentary layer through the surface. In the nearby regions (Fig. 1C) salt diapirs have already pierced to the surface where salt is rapidly removed by intense erosional processes and therefore, they do not manifest any longer as surface uplift. Inventories of surface deformation patterns related with salt diapirs are controlled by geologic factors (for example, type and thickness of overlying sediment layers), and the presence of tectonic stresses will likely significantly affect the inventories. Judging from the elliptical shape of the surface deformation (Fig. 2A) this can be attributed largely to a nonsymmetric salt diapir. Local tectonic stresses and geological heterogeneities within the sedimentary layer may be associated with a salt diapir, and create the surface deformation pattern as revealed by InSAR. Additionally, the thermal structure of the overlying sedimentary layer can modify the salt diapir evolution. Geological studies and seismic profiles suggest an initial relatively horizontal and continuous layering of the sedimentary deposits (Fig. S4)^42^. Therefore, critical to our understanding of a salt diapir evolution is the influence of tectonic history specific for our study area, as well as its rheological behavior. Particularly we are interested in taking the advantage of these InSAR observations which combined with numerical simulation can better constrain the salt diapir viscosity. To investigate how salt diapirs evolve from depth to surface, we employed two-dimensional high-resolution thermomechanical numerical models where we employ a Newtonian layer of salt overlain by a non-Newtonian layer of clastic sedimentary rocks (Supplementary Fig. S5). Since our numerical model is two-dimensional while the salt structures are naturally three-dimensional, our predictions should be considered as end member estimates. We decided to model the evolution (post Napee emplacement to present-day) since late Miocene because during the Napee emplacement stage the initial salt layer was likely transported horizontally to the east or southeast more than 100 km^42^. The numerical models are integrated some 4 Myr during the extensional period (from 9.3 to 5.3 Ma), followed by a compressional stage. Modelling results show that in the absence of tectonic processes the salt diapir is not able to pierce through the overlying sedimentary layers in the time frame considered in this study. This is consistent with previous studies where passive upward movement of salt takes long periods of time^50^. However, introducing episodes of extension (i.e. 4 Myr, from 9.3 to 5.3 Ma) followed by compression (Fig. 3), our modelling shows the formation of several large shear bands within the sedimentary layer (Supplementary SM 1). The combination of low strength, low viscosity, positive buoyancy of salt, and shear bands guide the successful propagation to surface of the salt diapir (Supplementary SM 2). The upward movement of a salt diapir takes some time (i.e. several Myr) to mobilize, but once it started to ascent this happens quite fast (within less than 1 Myr) (Supplementary SM 2). Approaching the surface, the salt diapir wall shaped started to produce a mushroom head that becomes more flattened when completely surface (Supplementary SM 3). Considering a wide range of parameters related with the initial 800 m salt layer viscosity (1 × 10^16^–1 × 10^19^ Pa s) and clastic sedimentary layer strength (i.e. cohesion 0.8–1.2 MPa), our modelling predicts different surface deformation gradients (Supplementary Fig. S6). Actually, we found a best fit model, in terms of both surface uplift rate and wavelength, for a salt viscosity of 1 × 10^17^ Pa s and a sedimentary layer cohesion of 1.1 MPa (Fig. 4). For smaller salt viscosities and low sedimentary rocks strength we obtain considerably higher surface uplift rates, including a very fast ascent of salt diapir, where the rising time until the salt reaches the surface happens only within the 4 Myr extensional period. Increasing more the salt viscosity and the sedimentary rocks strength, produces smaller surface deformation gradients, and for some upper values the salt diapir is rather trapped within the sedimentary layer and never reaches the surface (Supplementary Fig. S6). Decreasing the initial salt layer (i.e. 600 m) surface deformation is quite small and outside the InSAR observations. Our best model is obtained for a surface erosion rate of 0.2 mm/year^51,52^ and a transport distance of 2 kms. Models without erosion show a substantially higher (i.e. double) surface deformation gradients, whereas increasing both the erosion rate and the transport distance reduces the surface uplift rates (Supplementary Fig. S7). Also, in this model we use a background horizontal strain rate for extension period of 3.17 × 10^–16^ s^−1^ (± 0.25 mm/year) and of 6.34 × 10^–16^ s^−1^ (± 0.50 mm/year) for the shortening period, and increasing or decreasing these values affects direct proportionally (up to three times) the surface uplift rate (Supplementary Fig. S8). On the other hand, initial bottom temperature variations (190–220 °C) seem to play a rather limited effect on the salt diapir dynamics for our best fitting model (Supplementary Fig. S9).

In terms of deformation patterns, our modeling results show the formation on top of the salt diapir head of trap-like sedimentary structures (Fig. 4). These are consistent with the presence of several long-lived (more than 1 km deep (personal communication)) oil extraction wells located along the northern rim of the uplifted area (Fig. 2B,C).

In this study, using a combination of InSAR observations and robust numeric modelling we were able to constrain the viscosity of a relatively small rising salt diapir in southern Romania. Our results show that the effective propagation of weak and buoyant salt diapirs produces a surface signal in terms of uplift rates for a salt viscosity in the range of 1 × 10^17^ Pa s. Additionally, this novel combination of methods represents a cost-effective procedure that can be readily extended to other study regions for a faster identification and investigation of previously unexplored salt diapirs.

## Methods

### SBAS algorithm

For the purpose of detecting and characterizing the dynamic evolution of the earth surface in the DFZ study area between 2014 and 2018, we employ the Small BAseline Subset algorithm for analyzing the C-band SAR imagery acquired by the Sentinel-1A and B satellites. The data has a spatial resolution of 20 × 5 m and a signal wavelength of 5.6 cm, and it is made freely available by the European Space Agency. The SBAS and the Persistent Scatterer (PS) algorithms are the most popular multi-temporal interferometric techniques that were developed in the last 20 years^7,53^. Multi-temporal interferometry was proposed to improve the results obtained using simple differential interferometry by reducing the effects of the atmosphere, topography or large baselines. The multi-temporal approach increases the sensitivity of displacement detection from centimeters to millimeters. The main differences between the PS and the SBAS method consist in the types of targets detected. The PS method identifies discrete reflectors that present high coherence over the whole period of observation. Targets with stable signal are usually man-made structures and unvegetated rocks. The SBAS method on the other hand is based on the exploitation of multiple interferograms paired suitably according to the minimum temporal and spatial baselines between them. This technique offers the possibility to enhance coherence through reduction of the temporal and spatial decorrelation. Unlike PS methods, the SBAS exploits coherence over larger areas using Delaunay triangulation^54^ and can detect surface displacement for distributed scatterers with homogeneous characteristics, such as debris, desert or areas with short vegetation. Therefore, this method is more suitable for applications in the rural and natural areas that have less potential stable radar scatterers. Also, the method offers the capacity to derive quality results from smaller stacks of images, decreasing the computation demands which are usually significant for interferometric processing.

### Numerical model setup and boundary conditions

The numerical modelling was performed following the numerical technique of ref.^55^ to solve the 2D momentum, continuity and energy equations with the finite differences method. Our models also incorporate a depth-dependent, realistic non-Newtonian visco-plastic rheology for the clastic sedimentary layer, and a linear Newtonian rheology for the salt layer (Supplementary Eqs. (1)–(4)). In our numerical models plasticity is implemented using an yield criteria which limits the creep viscosity (Supplementary Eqs. (4), (5)). The creep viscosity of rocks is represented as a function of temperature and stress in terms of deformation invariants by experimentally determined flow laws (Supplementary Eqs. (6), (7), Supplementary Table S3). Numerical setup and boundary conditions are presented in detail in Fig. S5. The initial material setup involves a 7.2 km thick uniformly layered clastic sedimentary rock sequence, underlain by an 800 m thick salt layer placed at the bottom of the modeling domain (see Supplementary Table S3 for detailed material properties and rheology). For a wide range of salt viscosity (1 × 10^16^–1 × 10^19^ Pa s) numerical tests with a thinner initial layer (i.e. 600 m) produce surface deformation gradients that are small compared with surface uplift observed from InSAR. In order to facilitate diapir formation in the middle of the computational domain we include a small gaussian shape salt dome perturbation (2 km wide and 1 km in height) at the bottom of the model. The initial thermal structure is uniform with 0 °C at the surface and linearly increasing to 190–220 °C at the bottom of the model domain at 8 km below the clastic sedimentary layer upper surface. The temperature range used at the bottom of the model domain is also consistent with the thermal gradients (23–30 °C/km) reported in the study area by ref.^56^ and with the inverted geothermal profile of the central Moesian Platform of refs.^57,58^. The topographic evolution accounts for the effects of erosion and sedimentation. The clastic sediments/sticky-air interface evolves according to the transport Supplementary Eq. (4), which is solved at each time-step on the Eulerian grid. The model is extending and shortening with time according to the extensional Post-Nappe Emplacement and compressional Walachian deformation stages, and we use a constant extending rate of 0.45–0.55 mm/year (strain rates of 2.85–3.45 × 10^–16^ 1/s) followed by a constant shortening rate of 0.75–1.25 mm/year (strain rates of 4.76–7.93 × 10^–16^ 1/s)^22,47^. The top surface of the models represents an internal free surface through a 2 km thick layer of “sticky air”^55^. We use a viscosity cut-off lower and upper limits of 10^16^–10^19^ Pa s and 10^25^ Pa s respectively. For the “sticky air” layer we use fixed value of 10^18^ Pa s. The initial model size is 50 × 10 km, and we used an irregularly spaced numerical grid with a higher resolution of 250 × 100 m at the middle-top of the model and coarser resolution for the rest of the model. We use a viscoelastic numerical timestep of 10^3^ years. Lithology evolution through time is obtained by 120,000 randomly distributed Lagrangian tracers advected accordingly to the computed velocity field and a fourth-order Runge–Kutta scheme^55,59^.

## Supplementary Information

Supplementary Information.
