# Supplementary material for: InSAR surface deformation and numeric modeling unravel an active salt diapir in southern Romania
Source: Sci Rep. 2021 Jun 8;11:12091. doi: 10.1038/s41598-021-91517-4 (PMC8187355; doi:10.1038/s41598-021-91517-4)
Supplement: Supplementary file 1 — Supplementary Information. [file 41598_2021_91517_MOESM1_ESM.docx]

Supplementary Information for

**InSAR surface deformation and numeric modeling unravel an active salt diapir in southern Romania**

**Authors**: Vlad Constantin Manea^1,2,3^, Iuliana Armaş^4^, Marina Manea^1,2,4*^, and Mihaela Gheorghe^4,5^

^1^-Computational Geodynamics Laboratory, Centro de Geociencias, Universidad Nacional Autónoma de México, Campus Juriquilla, Querétaro, 76230, México.

^2^-Astronomical Institute of the Romanian Academy, 040557, Bucharest, Romania

^3^- Research Institute of the University of Bucharest–iCUB, University of Bucharest, 050095 Bucharest, Romania.

^4^-University of Bucharest, Faculty of Geography, Department of Geomorphology-Pedology-Geomatics, Nicolae Balcescu 1, Sector 1, 010041, Bucharest, Romania

^5^-GMV Innovating Solutions SRL, Calea Floreasca 246C, 077190, Bucharest, Romania

^*^Corresponding author M. Manea: marina@geociencias.unam.mx

**Contents of this file**

1. Topography and simplified geological maps corresponding to the Diapir Fold Zone (Supplementary Figure S1).

2. InSAR Data processing technique (Supplementary Figure S2, Figure S3)

3. Supplementary Tables 1, 2 and 3.

4. Geological cross-section **(**Supplementary Figure S4).

5. Numerical model settings **(**Supplementary Figure S5).

6. Modeling results. Supplementary Figure S6, Figure S7, Figure S8 and Figure S9.

7. Supplementary Movies SM1, Movie SM2 and Movie SM3.

8. Supplementary References.

1. In Supplementary Figure S1A we show the topography of the study area as color-shaded top-view map. In this figure tectonic faults^1^ are presented as red curves, and oil wells located in the vicinity of the Prahova and Doftana rivers confluence are shown as white round markers. In Supplementary Figure S1B we present a simplified top-view geological map of the Diapir Fold Zone draped over the topography^2,3^. Both maps were created based on ETOPO1 Global Relief Model dataset from ref. 4.

| 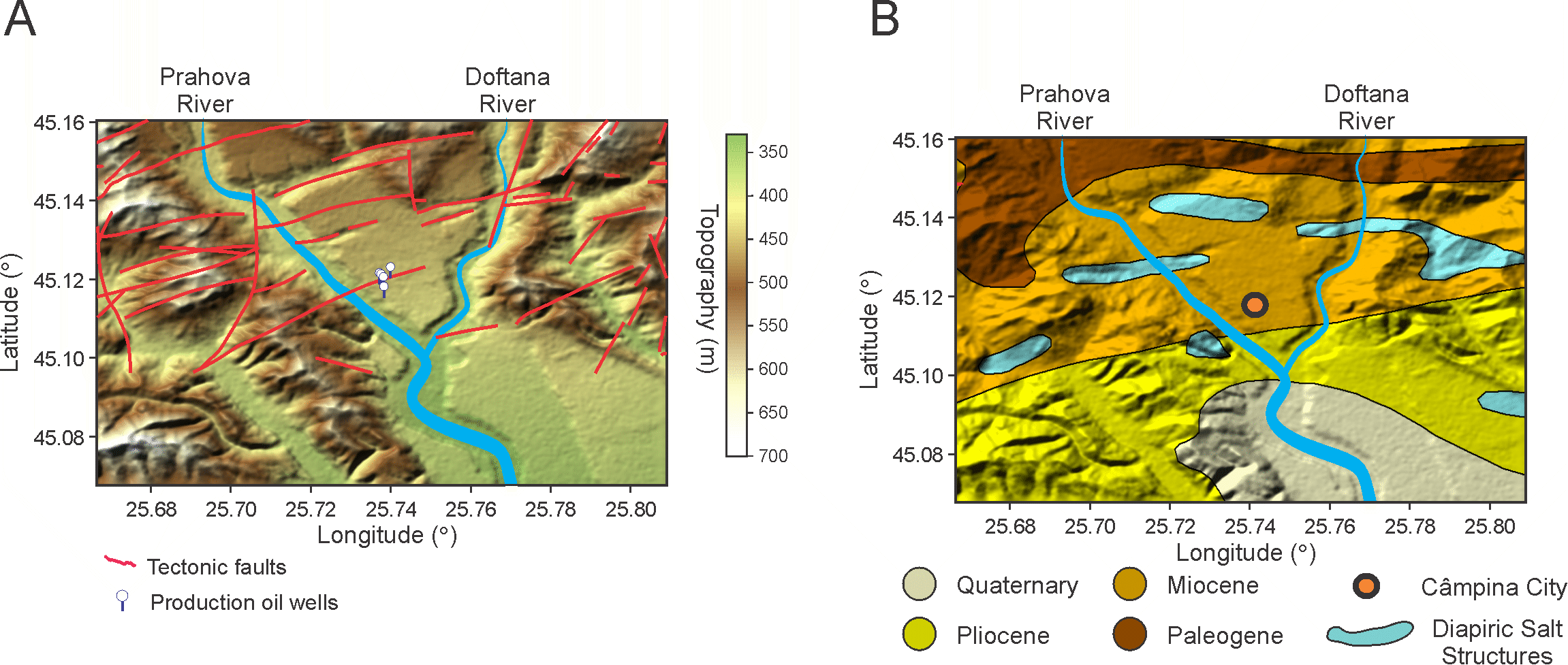 |
| --- |
| **Supplementary Figure S1. A:** Color-shaded top-view of the topography and tectonic faults for the study area^1^. White round markers show the location of production oil wells. **B:** Color-shaded top-view of the simplified geological map for the Diapir Fold Zone draped over the topography^2,3^. Maps are generated with the open-source software ParaView (http://www.paraview.org) version 5.0.1, licensed under the CC BY 4.0 license (<https://creativecommons.org/licenses/by/4.0/>). |

**2. InSAR Data processing**

Sentinel-1A and B satellites have acquired images over the study area from both, ascending and descending orbits, since the Sentinel-1A satellite became operational in October 2014. The stacks of images available at the Copernicus hub (https://scihub.copernicus.eu/) contain 62 Sentinel-1A and B images acquired from ascending orbit, on track 131, and 61 images acquired from descending orbit, on track 109. The temporal resolution of the data stacks is of approximately 1 month, and the Sentinel-1/B images parameters are presented in supplementary Table S1. The extents of areas selected from the entire Sentinel images are presented in Supplementary Figure S2.

| 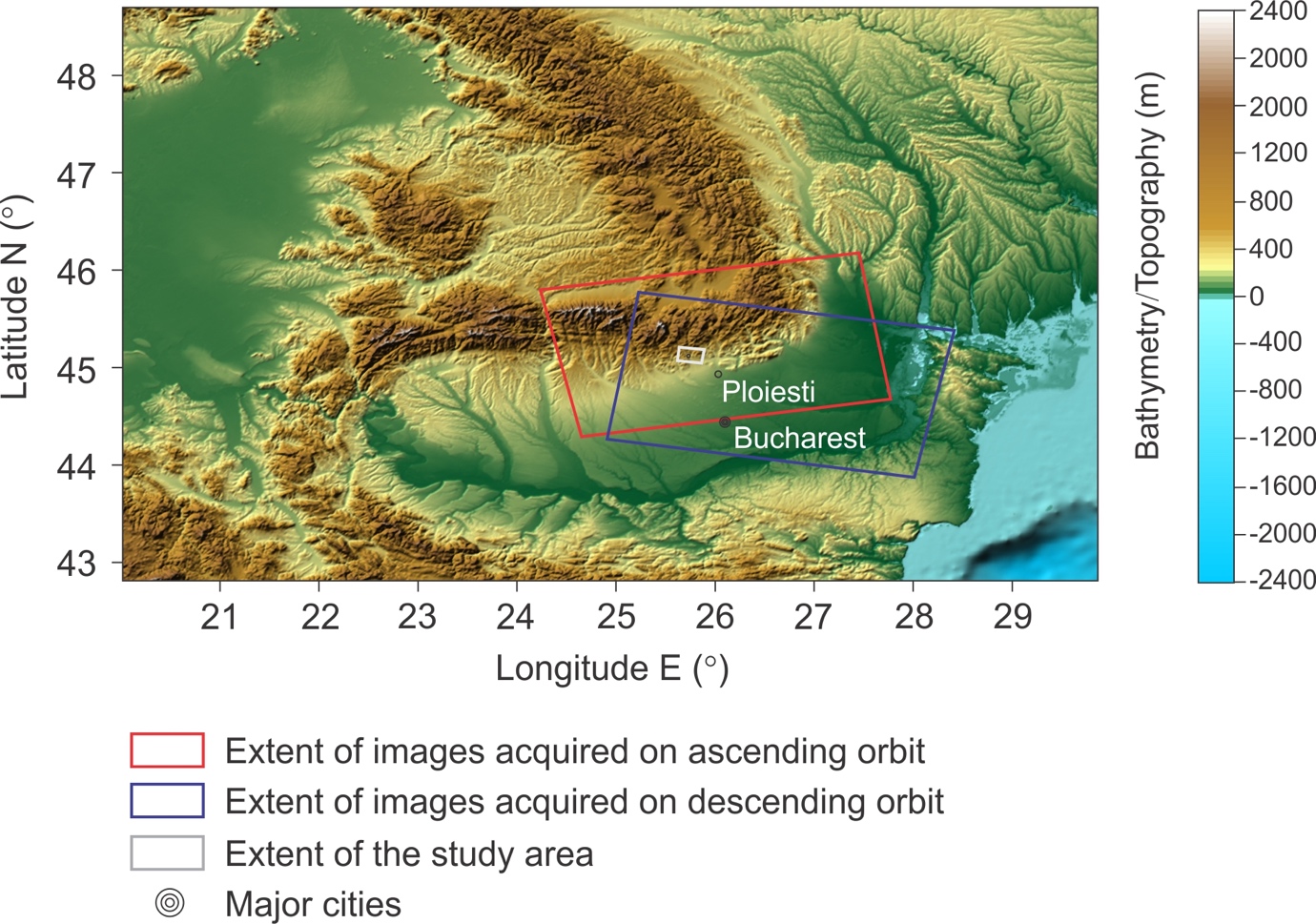 |
| --- |
| **Supplementary Figure S2.** Extent of the areas covered by the descending (blue square) and ascending (red square) acquisition orbit of the Sentinel-1A and Sentinel-B satellites. Extent of the study area is shown as small white square. The relative orbit numbers together with the localization of the area of interest provide an exact identification of the stacks of images that are used in the study. Map is generated with the open-source software ParaView (http://www.paraview.org) version 5.0.1, licensed under the CC BY 4.0 license (<https://creativecommons.org/licenses/by/4.0/>). |

The SBAS multi-temporal interferometric analysis was performed using the SARscape 5.4 Module for ENVI software provided by HARRIS Geospatial Solutions, USA. In order to minimize orbit inaccuracies, the latest precise orbit ephemerides provided by ESA for Sentinel-1 satellites were used for orbital correction. After this, interferograms were generated by pairing images with respect to multiple master images while imposing threshold values on temporal and spatial baselines. The temporal baseline threshold was set to 200 days and the spatial baseline maximum was set to 800 m. The goal was to reduce the signal decorrelation and the impact of uncorrected topography on the residual phase. After imposing the same constraints on both image stacks, 807 interferograms are obtained from the ascending stack and 865 interferograms from the descending stack respectively. All slave scenes were geo-referenced to the image acquired on 2 August 2016, which was established as a super-master due to being used as a master scene in most interferometric pairs. The spatial resolution of the images was resampled to 15 m by applying a multi-looking factor of 5 in azimuth and 1 in range. The effect of topography in the interferometric phase was removed using the SRTM 1 Arc-Second Global (30 meters) digital elevation model. The noise in the differential interferograms was removed with a Goldstein filter with window size of 18 pixels. Afterwards, the intermediate results (wrapped interferograms and unwrapped phases) were manually inspected to identify and discard those affected by strong inaccuracies caused by orbital effects that could not be corrected, very low coherence, atmospheric errors and residual topography. Thirty-two interferograms were discarded from the ascending stack and 45 interferograms from the descending stack. The interferograms were refined and flattened based on 26 ground control points arbitrary defined in flat areas that presented an unwrapped phase close to zero. In the last step, the residual height and velocity are estimated using both linear and quadratic inversion models for detecting potential non-linear movement trends (Supplementary Table S2).

Displacement over the study area display an uplift rate of up to 6 mm/year (Fig. S3)

| 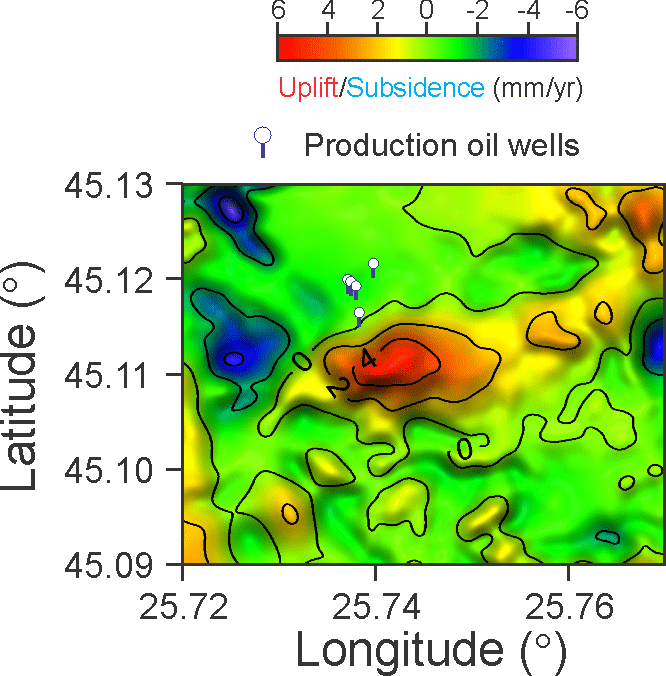 |
| --- |
| **Supplementary Figure S3.** Color-shaded top-view of the interpolated InSAR vertical displacement rates. White round markers show the location of several production oilrigs (maximum drilling depth of 1 km (personal communication)). |

**3. Supplementary Tables 1, 2 and 3.**

**Table S1**. Sentinel-1/B images parameters used in this study.

| **Orbit** | **Ascending** | **Descending** |
| --- | --- | --- |
| Number of observations | 62 | 61 |
| Time period | 14.10.2014-17.10.2018 | 13.10.2014-28.10.2018 |
| Incidence angle | 39.5^0^ | 39.4^0^ |
| Azimuth | 350^0^ | 190^0^ |

**Table S2.** Parameters provided in this study using SBAS multi-temporal interferometric analysis.

| **Attribute** | **Description** | **Unit** |
| --- | --- | --- |
| Point | Point unique ID |  |
| Velocity | estimated movement rate (average velocity) | mm/year |
| Coherence | coherence (quality, precision measure) |  |
| H_Precision | standard deviation of the relative height | m |
| V_Precision | standard deviation of the subsidence rate | mm/year |
| Lon | point coordinates (geographic longitude) | ° |
| Lat | point coordinates (geographic latitude) | ° |
| X, Y, Z | cartesian point coordinates | m |
| Los_Az | angle between satellite line of sight (LOS) and Azimuth direction | ° |
| Los_In | LOS incidence angle | ° |
| Height_cor | correction of the relative height | m |
| Tot_Displ | cumulative displacement between first and last acquisition | mm |
| D_yyyymmdd | displacement between first and current acquisition | mm |

**Table S3. Material properties used in numerical experiments.**

| **Material** | **Density**  **(kg/m^3^)** | **Thermal conductivity**  **(W/m K)** | **Rheology**  **Ea (kJ/mol), A_D_ (MPa^-n^s^-1^), c (MPa)** | **Heat capacity**  **(J/kg K)** |
| --- | --- | --- | --- | --- |
| Sticky Air Layer | 1000 (solid) (ref. 5) | 300 (ref. 5) | Constant viscosity: 10^18^ Pa s  (ref. 5) | 300  (ref. 5) |
| Clastic sedimentary rocks | 2600 (solid)  (ref. 6) | 0.64+807/(T-77); where T is temperature in (K). (ref. 9) | Power flow law, n=2.3, Ea=154, A_D_=3.2E-4, c=0.6-1.2 (this study), φ=11.54° (sin(φ)=0.2) (ref. 9) | 1000  (ref. 5) |
| Salt (halite) | 2160 (solid)  (ref. 7) | 6.5  (ref. 8) | Newtonian flow law, n=1, Ea=24.53, A_D_=4.7E-4 (ref. 10), c=4, φ=30° (sin(φ)=0.5) (ref. 11) | 916  (ref. 11) |

Table 3 (continuation)

| **Material** | **Shear Modulus**  **(Pa)** | **Thermal expansion (1/K)** | **Compressibility**  **(1/Pa)** | **Radioactive heating (μW/m^3^)** | **Viscosity cut-offs (Pa s)** |
| --- | --- | --- | --- | --- | --- |
| Sticky Air Layer | 1x10^20^  (ref. 5) | 3x10^-5^  (ref. 5) | 1x10^-11^  (ref. 5) | 0 (ref. 5) | Fixed value: 10^18^ (ref. 5) |
| Clastic sedimentary rocks | 1x10^10^  (ref. 5) | 3x10^-5^  (ref. 5) | 1x10^-11^  (ref. 5) | 2  (ref. 7) | Upper limit: 10^25^ (ref. 5) |
| Salt (halite) | 1.261x10^10^  (ref. 7) | 3x10^-5^  (ref. 7) | 1x10^-11^  (ref. 7) | 0.25  (ref. 7) | Lower limit: 10^16^-10^19^ (this study) |

4. **Geological cross-section.**

This geological cross section is based on the seismic-reflection section^12^.

| 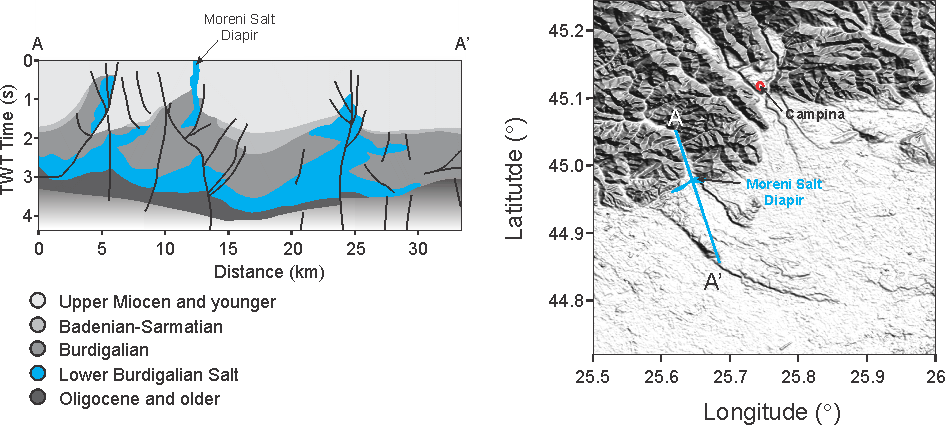 |
| --- |
| **Supplementary Figure S4. Left:** Geological cross-section A-A’ through the Moreni salt diapir (blue color). Figure adapted from ref. 12 and ref. 13. Note the presence of a bottom continuous (TWT <0.5 s) salt layer that feeds several diapirs. **Right:** The cross-section A-A’ is located ~15 km SW from our study area. Map is generated with the open-source software ParaView (http://www.paraview.org) version 5.0.1, licensed under the CC BY 4.0 license (<https://creativecommons.org/licenses/by/4.0/>). |

**5. Numerical model settings**

In our 2-D thermo-chemical numerical model the materials are treated as a fluid with visco-elasto-plastic rheology^14,15,16,17,18^, where the total strain-rate ${(\dot{\varepsilon}}_{ij}$) of the visco-elasto-plastic fluid is computed as following:

$\dot{\varepsilon}_{ij}$=$\dot{\varepsilon}_{ij(elastic)}+ \dot{\varepsilon}_{ij(viscouss)}+ \dot{\varepsilon}_{ij(plastic)}$ (1)

Where:

$\dot{\varepsilon}_{ij(elastic)}=\frac{1}{2\mu}\frac{D\dot{\sigma}_{ij}}{Dt}$ (2)

$\dot{\varepsilon}_{ij(viscouss)}=\frac{1}{2\eta}\dot{\sigma}_{ij}$ (3)

$\dot{\varepsilon}_{ij\left( plastic \right)}=0 for \sigma_{II}<\sigma_{yield}$ (4.1)

$\dot{\varepsilon}_{ij\left( plastic \right)}= \chi\frac{\dot{\sigma}_{ij}}{2\sigma_{II}} for \sigma_{II}=\sigma_{yield}$ (4.2)

$\sigma_{II}=\sqrt{\frac{{\dot{\sigma}_{ij}}^{2}}{2}}$ and $\sigma_{yield}=c+\sin\varphi*P$ (5)

Where $\dot{\varepsilon}_{ij}$ is the strain rate, *η* the viscosity, and *μ* the shear modulus. $\frac{D\dot{\sigma}_{ij}}{Dt}$ is the objective co-rotational time derivative of the deviatoric stress component $\dot{\sigma}_{ij}$, *μ* is the…, $\sigma_{II}$is the second invariant of the deviatoric stress tensor, and *χ* is the plastic multiplier (unknown a priori), which satisfies the plastic yielding condition $\sigma_{II}=\sigma_{yield}$. *c* is the rock cohesion, $\varphi$ is the internal angle of friction (Supplementary Table S3) and *P* pressure.

The rheological flow law used is:

$\dot{\varepsilon_{II}}=A_{D}{(\sigma_{II})}^{n}e^{(-\frac{E_{a}}{RT})}$ (6)

And the viscosity for diffusion creep is computed as:

$\eta_{diff}=\frac{1}{2}A_{D}\sigma_{ij}^{1-n}e^{(\frac{E_{a}}{RT})}$

(7)

where the experimental flow law parameters are presented (Supplementary Table S3).

The numerical calculations are performed by solving the conservation equations of mass, momentum and energy, considering both radioactive and shear heating terms^14^.

The computational domain contains two rock type layers, clastic sediments and salt (halite). The sedimentary layer has a uniform composition, and the dark and light brown stripes are used to better highlight the temporal deformation of this layer (Supplementary Figure S5). We include on top of the sedimentary layer a “sticky-air” layer with a thickness of 2 km to allow self-consistent surface deformation^19,20,21^. The interface between the “sticky-air” layer and the clastic sediments is affected by erosion and sedimentation^22^. This surface evolves according to the following equation (solved for each time step)^20^:

$\frac{{\partial y}_{es}}{\partial t}=v_{y}-v_{x}\frac{\partial y_{es}}{\partial x}+\frac{\partial}{\partial x}\left( K_{s}\frac{\partial y_{es}}{\partial x} \right)$ (8)

$K_{s}=\frac{e_{r}L_{t}^{2}}{h_{max}}$ (9)

Where *y_es_* is the vertical position of the surface, *v_y_* and *v_x_* are the vertical and horizontal components of the material velocity vector at the surface, and *K_s_* is the effective ‘topography diffusion’ coefficient, $e_{r}$is the erosion rate (0.1-0.3 mm/yr)^23,24^, *L_t_* is the transport lenghtscale (2-10 km), and *h_max_* is the maximum elevation (2 km).

**Supplementary Figure S5. Initial numerical model setup for the two-dimensional salt model.**

Here we show the mode geometry and boundary conditions used in the modeling. The initial rectangular box is 50 km x 10 km, and no material can flow in or out of the modeling domain. We use a nonuniform grid, with a maximum resolution of 250 m x 100 m in the top central region. The initial temperature distribution is defined by linear geotherm with a bottom constant temperature between 190^0^C and 220^0^C, which corresponds to a geotherm within the thermal gradient range of 23-30^0^C/km reported in the study area^25^. We use a three rock type layers in the model, the top 2 km sticky weak layer has a prescribed low viscosity of 10^18^ Pa s, a clastic sedimentary rock layer with a non-Newtonian rheology, and a bottom slat layer with a linear Newtonian rheology. We used a salt viscosity cut-off in the range of 1x10^16^ – 1x10^19^ Pa s. We prescribed an initial *h_salt_* of 800 m (a series of numerical tests with a thinner (i.e. 600 m) salt layer is also performed) (*ρ_salt_*=2160 kg/m^3^) with an initial central gaussian shape anomaly (2 km wide and 1 km in height). The bottom salt layer is shown in blue, and the sedimentary layer is shown as a bi-tone brown color multiple layer structure. Half the velocity magnitude is applied in opposite directions at both ends of the model. Lateral velocity is prescribed orthogonal to the boundaries and a compensating outward/inward velocity is prescribed on the bottom of the model in order to insure mass conservation. Material physical and rheological values used in our model are presented in Table S3.

| 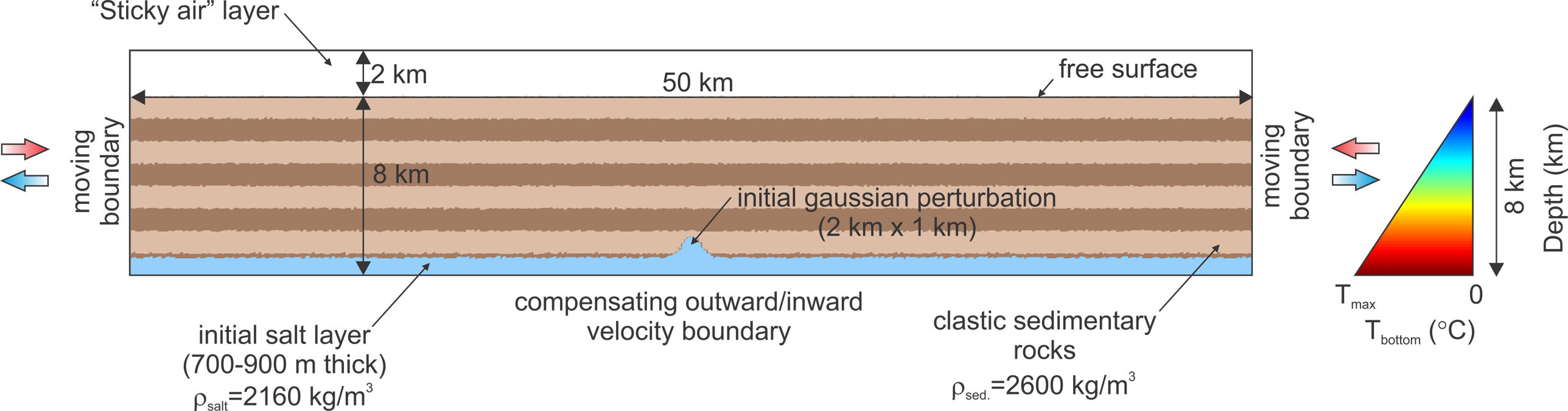 |
| --- |
| **Supplementary Figure S5.** Model setup, initial and boundary conditions. |

**6. Supplementary figures for modeling results (S6-S9)**

In this section we present the modeling sensitivity tests in form of parameter tables, where we compare the surface deformation gradient from numerical modeling with the uplift rates inferred from InSAR data analysis. The first supplementary figure (supplementary figure S6) shows the effect of slat layer viscosity, initial layer thickness, and clastic sediments cohesion on the surface deformation gradient. In the subsequent supplementary figures, we test our best model (see the gray shaded square in supplementary figure S6) against different erosion rates (0-0.3 mm/yr) and transport length scales (0-6 km) (supplementary figure S7), background horizontal strain rates for extension (2.85 x10^-16^ - 3.49x10^-16^ s^-1^) applied for 4 Myr, and shortening (4.76 x10^-16^ – 7.93x10^-16^ s^-1^) applied for the rest of the simulation (supplementary figure S8). The bottom temperature tests (190-220^0^C) are shown in supplementary figure S9.


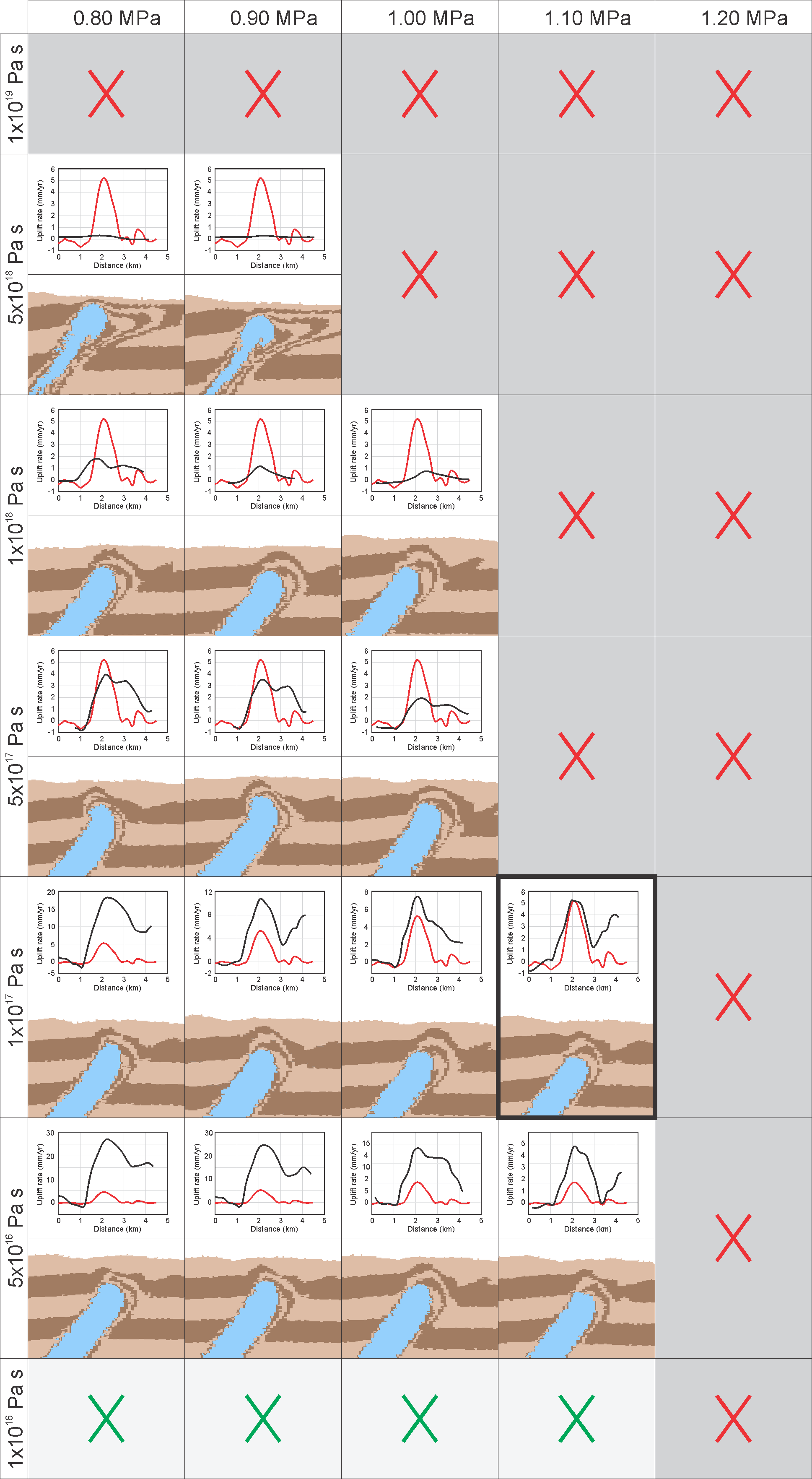


**Supplementary Figure S6. Image illustrating the surface deformation gradient (black curves) vs. InSAR observation (red curves) for different salt layer viscosities (1x10^16^-1x10^19^ Pa s) and clastic sediment layer cohesions (0.8-1.2 MPa).** In all these numerical experiments the background horizontal strain rate for extension period is 3.17e^-16^ s^-1^ (±0.25 mm/yr) and 6.34e^-16^ s^-1^ (±0.5 mm/yr) for the shortening period. The initial salt layer thickness is set to 800 m, and the initial bottom temperature is 200^0^C. The best fitting model is marked with a black square and corresponds to a salt viscosity of 1x10^17^ Pa s. Regions marked with a red cross represent models where the salt diapir did not reach the surface during the simulation, and regions with a green cross represent regions where the salt diapir arrived at the surface very fast during the extension period only. The rocks distribution snapshot shown below each plot represents a 7 km wide zoom in centered on the salt diapir head.

**
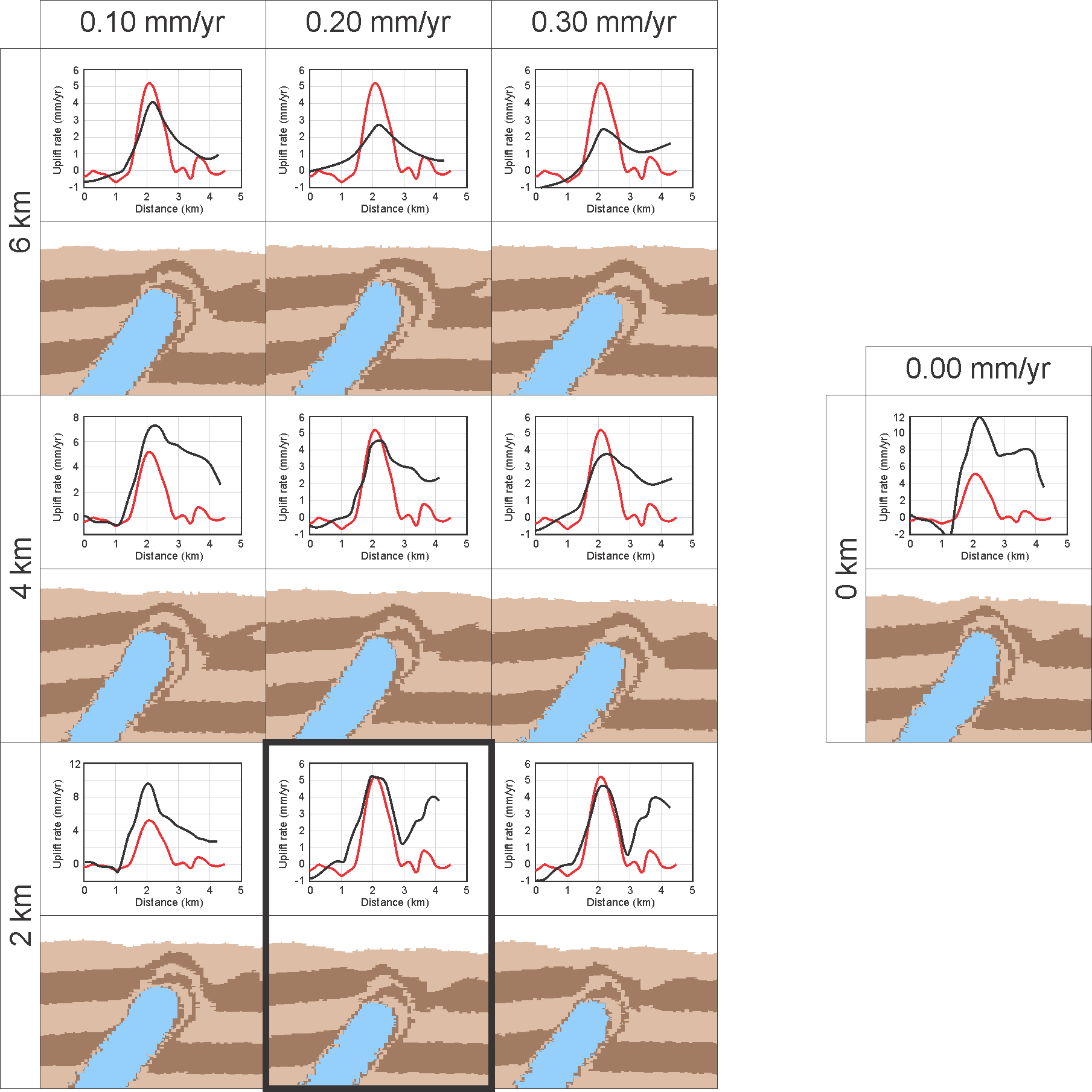
**

**Supplementary Figure S7. Image illustrating the surface deformation gradient (black curves) vs. InSAR observation (red curves) for different surface erosion rates (0-0.3 mm/yr) and transport length scales (0-6 km).** In all these numerical experiments we use parameters specific for the best fitting model marked with a black square: the background horizontal strain rate for extension period is 3.17e^-16^ s^-1^ (±0.25 mm/yr) and 6.34e^-16^ s^-1^ (±0.50 mm/yr) for the shortening period; the initial salt layer thickness is set to 800 m; the clastic sediments cohesion is 1.1 MPa; the initial bottom temperature is 200^0^C, and the salt viscosity is 1x10^17^ Pa s. The righthand model in performed in the absence of surface erosion. The rocks distribution snapshot shown below each plot represents a 7 km wide zoom in centered on the salt diapir head.


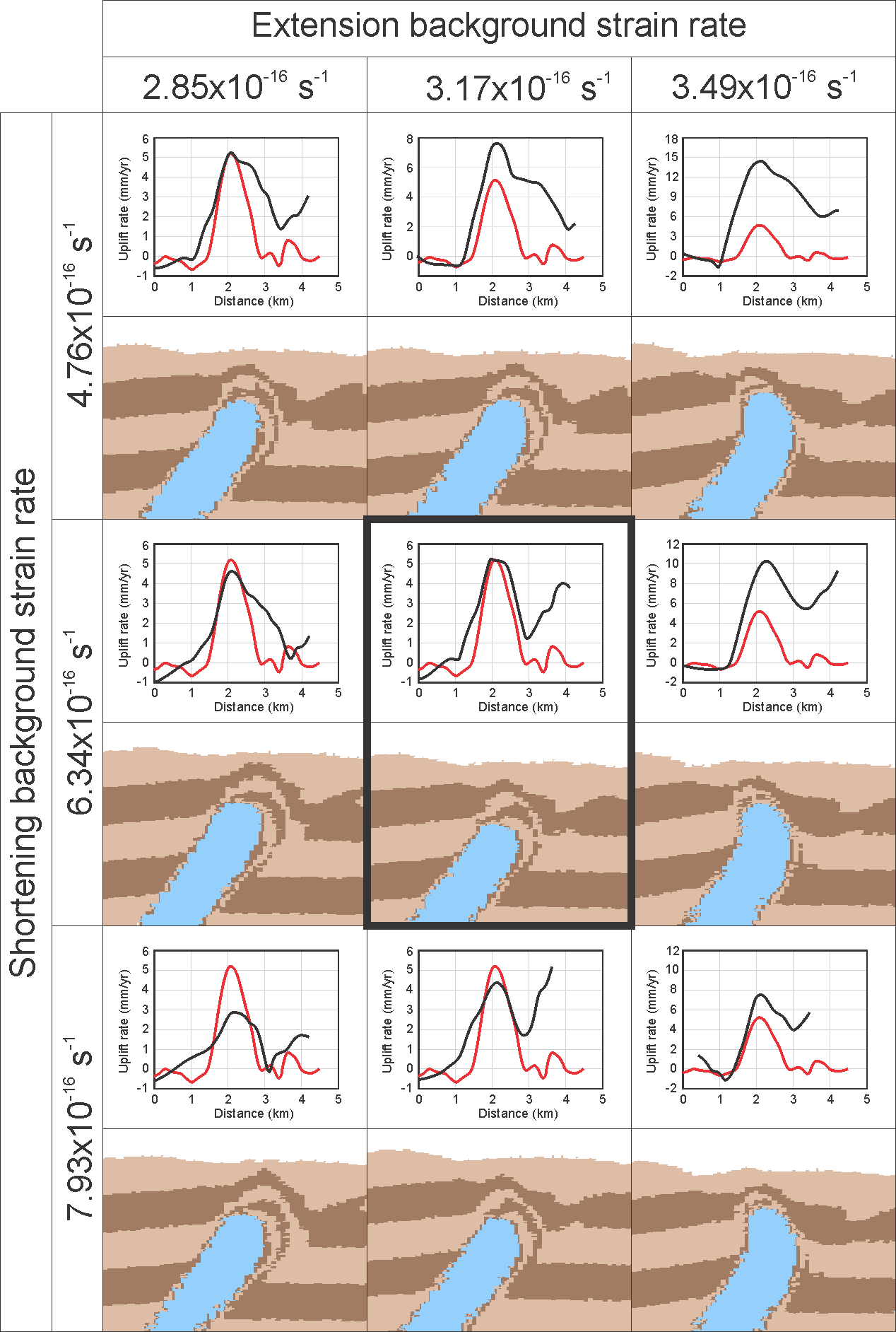


**Supplementary Figure S8. Image illustrating the surface deformation gradient (black curves) vs. InSAR observation (red curves) for different background horizontal strain rates.** The best fitting model is marked with a black square. All other parameters are taken for our best model: the initial salt layer thickness is set to 800 m; the clastic sediments cohesion is 1.1 MPa; the initial bottom temperature is 200^0^C, and the salt viscosity is 1x10^17^ Pa s. The rocks distribution snapshot shown below each plot represents a 7 km wide zoom in centered on the salt diapir head.


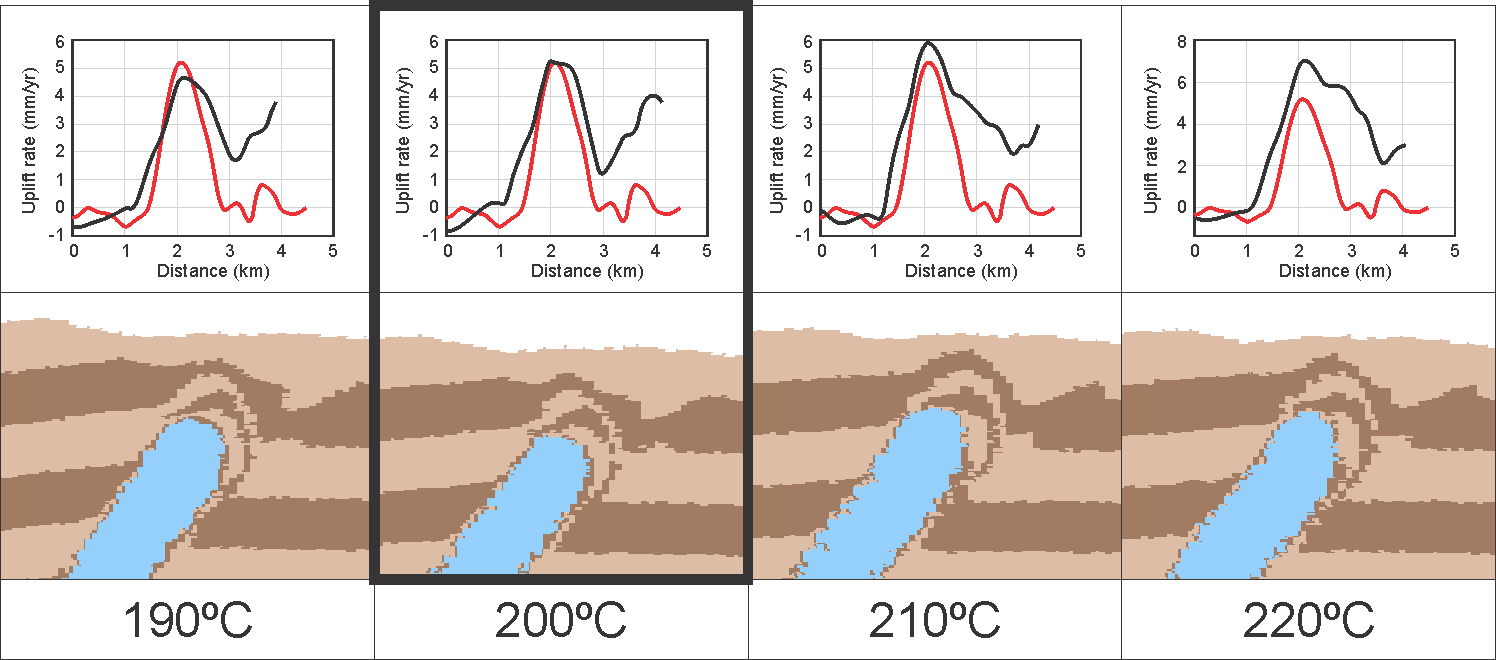


**Supplementary Figure S9. Image illustrating the surface deformation gradient (black curves) vs. InSAR observation (red curves) for different initial bottom temperatures (190-220**^0^**C).** All other parameters are for our best model (marked with a black square): the background horizontal strain rate for extension period is 3.17e^-16^ s^-1^ (±0.25 mm/yr) and 6.34e^-16^ s^-1^ (±0.50 mm/yr) for the shortening period; the initial salt layer thickness is set to 800 m; the clastic sediments cohesion is 1.1 MPa, and the salt viscosity is 1x10^17^ Pa s. The rocks distribution snapshot shown below each plot represents a 7 km wide zoom in centered on the salt diapir head.

**7. Supplementary movies**

**Supplementary movie SM1. Animation showing the evolution of temperature, viscosity and density for the numerical experiment which is considered our best fit model (see Supplementary Figure S6).** In this numerical experiment we use a background horizontal strain rate for extension period is 3.17e-16 s-1 (±0.25 mm/yr) and 6.34e-16 s-1 (±0.5 mm/yr) for the shortening period. The initial salt layer viscosity is 1x10^17^ Pa s, a thickness of 800 m, and an initial bottom temperature of 200°C. In this animation the depth scale is relative to the top of the sticky-air layer.

**Supplementary movie SM2. Animation showing the evolution of stresses and second invariant of the deviatoric strain rate tensor for the numerical experiment which is considered our best fit model (see Supplementary Figure S6).** The numerical experiment parameters are as specified in supplementary animation SM1. In this animation the depth scale is relative to the top of the sticky-air layer.

**Supplementary movie SM3. Animation showing the evolution of rocks for the numerical experiment which is considered our best fit model (see Supplementary Figure S6).** The numerical experiment parameters are as specified in supplementary animation SM1. In this animation the depth scale is relative to the top of the sticky-air layer.

**8. Supplementary References.**

1. Murgeanu, G., Motas. I., Bandradur, T., Ghenea, C. & Sandulescu, M. Geological Map of Romania, scale 1:200000, sheet no. 36-Ploiesti: Geological Institute of Romania, Bucharest (1967).

2. Tămaș, D.M., Schléder, Z., Krézsek, C., Man, S., and Filipescu, S. Understanding salt in orogenic settings: the evolution of ideas in the Romanian Carpathians. AAPG Bulletin, 102(6), p. 941-958 (2018).

3. Paraschiv, D. & Olteanu, G. Oil fields in mio-pliocene zone of Eastern Carpathians (District of Ploiești), in M. T. Halbouty, ed.: AAPG Memoir, no. 14, pp. 399-427. (1970).

4. Amante, C. & Eakins, B. W. ETOPO1 1 arc-minute global relief model: procedures, data sources and analysis (p. 19). Colorado: US Department of Commerce, National Oceanic and Atmospheric Administration, National Environmental Satellite, Data, and Information Service, National Geophysical Data Center, Marine Geology and Geophysics Division (2009).

5. Gerya, T. Introduction to numerical geodynamic modelling. Cambridge University Press, 484 pp. (2019).

6. Turcotte, D.L. & Schubert, G. Geodynamics 2nd edition. Cambridge University Press, 465 pp. (2002).

7. Jackson, M.P.A. & Hudec. M.R. Principles and Practice. Cambridge University Press, 498 pp. (2017).

8. Clauser, C. & Huenges, E. Thermal conductivity of rocks and minerals, in: T.J. Ahrens (Ed.), Rock Physics and Phase Relations, AGU Reference Shelf 3, AGU, Washington,DC, pp 105–126 (1995).

9. Ranalli, G., Rheology of the Earth, 2nd edition, Chapman and Hall, London, 413 pp (1995).

10. Spiers CJ, Schutjens PMTM, Brzesowsky RH, Peach CJ, Liezenberg JL, Zwart HJ. Experimental determination of constitutive parameters governing creep of rocksalt by pressure solution. In: Knipe RJ, Rutter EH, editors. Deformation mechanisms, rheology and tectonics. Geological Society, London, Special Publications. 54(1):215–27, (1990).

11. Kolano M. and Flisiak D. Comparison of geo-mechanical properties of white rock salt and pink rock salt in Kłodawa salt diapir. Studia Geotechnica et Mechanica. Vol. XXXV, No. 1, (2013).

12. Stefanescu M., Dicea O. & Tari G. Influence of extension and compression on salt diapirism in its type area, East Carpathians Bend area, Romania, in Vendeville, B., Marx, Y. & Vigneresse, J.-L. (eds) Salt, Shale and Igneous Diapirs in and around Europe. Geological Society, London, Special Publications, 174, p.131-147, (2000).

13. Schleder, Z., Tamas, D.M., Krezsek, C. et al. Salt tectonics in the Bend Zone segment of the Carpathian fold and thrust belt, Romania. Int J Earth Sci (Geol Rundsch) 108, 1595–1614 (2019).

14. Gerya, T. V. and D. A. Yuen. Robust characteristics method for modelling multiphase visco-elasto-plastic thermo-mechanical problems. Physics of the Earth and Planetary Interiors 163(1-4): 83-105, (2007).

15. Fuchs, L., Schmeling, H. and Koyi, H., Numerical models of salt diapir formation by down‐building: the role of sedimentation rate, viscosity contrast, initial amplitude and wavelength. Geophysical Journal International, 186: 390-400, (2011).

16. Fernandez, N., J.P. Kaus, B., Fold interaction and wavelength selection in 3D models of multilayer detachment folding, Tectonophysics, Volume 632, Pages 199-217, ISSN 0040-1951, (2014).

17. Fernandez, N., J.P. Kaus, B., [Pattern formation in 3-D numerical models of down-built diapirs initiated by a Rayleigh–Taylor instability](javascript:void(0)), Geophysical Journal International 202 (2), 1253-1270, (2015).

18. N Fernandez, MR Hudec, CAL Jackson, TP Dooley, OB Duffy, [The competition for salt and kinematic interactions between minibasins during density-driven subsidence: observations from numerical models](javascript:void(0)). Petroleum Geoscience 26 (1), 3-15, (2020).

19. Crameri, F., Tackley, P. J., Meilick, I., Gerya, T. V., & Kaus, B. J. P. A free plate surface and weak oceanic crust produce single‐sided subduction on Earth. Geophysical Research Letters, 39(3), L03306 (2012).

20. Gerya, T., Introduction to numerical geodynamic modelling. Cambridge University Press. 2^nd^ Edition. (2019).

21. Schmeling, H., A. Y. Babeyko, A. Enns, C. Faccenna, F. Funiciello, T. Gerya, G. J. Golabek, S. Grigull, B. J. P. Kaus, G. Morra, S. M. Schmalholz and J. van Hunen. A benchmark comparison of spontaneous subduction models-Towards a free surface. Physics of the Earth and Planetary Interiors 171(1-4): 198-223, (2008).

22. Gerya, T. V. and D. A. Yuen. Characteristics-based marker-in-cell method with conservative finite-differences schemes for modeling geological flows with strongly variable transport properties. Physics of the Earth and Planetary Interiors 140(4): 293-318, (2003).

23. Sanders, C. A. E., Andriessen, P. A. M., and Cloetingh, S.A.P.L., Life cycle of the East Carpathian orogen: Erosion history of a doubly vergent critical wedge assessed by fission track thermochronology, J. Geophys. Res., 104, B12, 29 095–29 112, (1999).

24. Bertotti, G., L. Matenco, and S. Cloetingh, Vertical movements in and around the SE Carpathian foredeep: Lithospheric memory and stress field control, Terra Nova, 15, 299 – 305, (2003).

25. Andreescu, M., Burst, D., Demetrescu, C., Ene, M., & Polonic, G. On the geothermal regime of the Moesian Platform and Getic Depression. Tectonophysics, 164(2–4), p. 281-286, (1989).
